# Complement Proteins C5/C5a, Cathepsin D and Prolactin in Chondrocytes: A Possible Crosstalk in the Pathogenesis of Osteoarthritis

**DOI:** 10.3390/cells11071134

**Published:** 2022-03-28

**Authors:** Sandeep Silawal, Miriam Wagner, Dominik Roth, Thomas Bertsch, Silke Schwarz, Maximilian Willauschus, Markus Gesslein, Jakob Triebel, Gundula Schulze-Tanzil

**Affiliations:** 1 Institute of Anatomy and Cell Biology, Paracelsus Medical University, Nuremberg, 90419 Nuremberg, Germany; sandeep.silawal@klinikum-nuernberg.de (S.S.); schwarzbiggy@yahoo.de (S.S.); 2 Faculty of Applied Chemistry, Nuremberg Institute of Technology Georg Simon Ohm, 90489 Nuremberg, Germany; miriam-w-94@web.de (M.W.); dominik.roth1994@t-online.de (D.R.); 3 Institute for Clinical Chemistry, Laboratory Medicine and Transfusion Medicine, Nuremberg General Hospital and Paracelsus Medical University, 90419 Nuremberg, Germany; thomas.bertsch@klinikum-nuernberg.de; 4 Department of Orthopedics and Traumatology, Nuremberg General Hospital and Paracelsus Medical University, 90471 Nuremberg, Germany; maximilian.willauschus@klinikum-nuernberg.de (M.W.); markus.gesslein@klinikum-nuernberg.de (M.G.)

**Keywords:** osteoarthritis, complement, C5, anaphylatoxin C5a, prolactin, prolactin receptor, cathepsin D

## Abstract

Introduction: Both increased activity of the complement system (CS) and the role of the pituitary hormone prolactin (PRL) are implicated in osteoarthritis (OA) pathogenesis. Besides, Cathepsin D (CatD) activity is increased in the context of OA and can exert not only proteolytic but also non-proteolytic effects on cells. For the first time, possible crosstalk between two separate humoral systems: the CS and the PRL hormone systems in chondrocytes are examined together. Methods: Primary human articular chondrocytes (hAC) were stimulated with complement protein C5 (10 µg /mL), PRL (25 ng/mL), CatD (100 ng/mL), or anaphylatoxin C5a (25 ng/mL) for 24 h or 72 h, while unstimulated cells served as controls. In addition, co-stimulations of C5 or PRL with CatD were carried out under the same conditions. The influence of the stimulants on cell viability, cell proliferation, and metabolic activity of hAC, the chondrosarcoma cell line OUMS-27, and endothelial cells of the human umbilical cord vein (HUVEC) was investigated. Gene expression analysis of C5a receptor (C5aR1), C5, complement regulatory protein CD59, PRL, PRL receptor (PRLR), CatD, and matrix metal-loproteinases (MMP)-13 were performed using real-time PCR. Also, collagen type (Col) I, Col II, C5aR1, CD59, and PRL were detected on protein level using immunofluorescence labeling. Results: The stimulation of the hAC showed no significant impairment of the cell viability. C5, C5a, and PRL induced cell growth in OUMS-27 and HUVEC, but not in chondrocytes. CatD, as well as C5, significantly reduced the gene expression of CatD, C5aR1, C5, and CD59. PRLR gene expression was likewise impaired by C5, C5a, and PRL+CatD stimulation. On the protein level, CatD, as well as C5a, decreased Col II as well as C5aR1 synthesis. Conclusions: The significant suppression of the C5 gene expression under the influence of PRL+CatD and that of CD59 via PRL+/−CatD and conversely a suppression of the PRLR gene expression via C5 alone or C5a stimulation indicates an interrelation between the two mentioned systems. In addition, CatD and C5, in contrast to PRL, directly mediate possible negative feedback of their own gene expression.

## 1. Introduction

### 1.1. Osteoarthritis

Osteoarthritis (OA) is the most common musculoskeletal disease of the modern world [1,2]. OA is not only a degenerative disease of the cartilage that is exclusively caused by excessive mechanical loading of the joint; various other predisposing factors such as aging, joint injury, genetics, anatomic malalignment of the joints in lower extremities also play their roles in the pathogenesis of OA [1]. All tissues in a joint such as cartilage, bones, synovial tissue, fat tissue, ligaments, and vessels are involved in the genesis of OA [3], where the tissues present a low-grade chronic inflammation leading to the ultimate degeneration of the whole joint [4]. Defining OA as an inflammatory disease, it becomes more and more obvious that the pro- and anti-inflammatory cytokines, hormones, immune activated cells, complement proteins, enzymes, etc. intertwine to affect the above-mentioned tissues resulting in the net degrading effect leading to OA [5,6,7]. Notably, inflammatory cytokines such as Interleukin-1β and tumor necrosis factor α are well-known candidates involved in the pathogenesis of OA [8]. These cytokines were detected in higher concentrations at the superficial layer in contrast to the deeper layer or the macroscopic healthy areas of the joint [8]. The CS as an intrinsic part of our immune system has been brought into focus in context with OA pathogenesis [9]. Even though cartilage is an avascular, aneural, and alymphatic tissue, angiogenesis is a typical feature associated with chronic OA [10,11]. Hence, understanding the effects of the cytokines and complement factors in angiogenesis of the cartilage could also help in understanding more about the progressive stages of the OA.

### 1.2. The Complement System

CS is a part of the innate immune system. The CS involves more than 30 proteins which are activated as a cascade through basically 3 pathways; the classical, the lectin, and the alternative pathway [12]. However, in recent years various other complement activating factors have been introduced, such as thrombin [13], plasmin [14] and the lysosomal enzyme CatD [15], which activate the CS in various stages of the cascade. This comprises the fragmentation of complement proteins such as C3, C4, and C5, generating the anaphylatoxins C3a, C4a, and C5a, respectively [12]. The anaphylatoxins lead to inflammation and chemoattraction of the leukocytes, which could, in turn, upregulate the ongoing low-grade inflammation in OA. As known, there is higher expression and activation of CS in human OA joints than in normal joints [16]. Complement regulatory proteins (CRP) are membrane-bound as well as soluble proteins that regulate the activated complement factors at different cascade levels keeping their own functional body cells intact [12]. The Membrane Attack Complex (MAC) is built up in the terminal phase of the complement cascade, where subunits C5b-C9 are associated with forming this complex on the cell membrane. It can either osmotically eliminate the unwanted cells or introduce inflammation and apoptosis. As one of the CRPs, CD59 hinders the assembly of the MAC, subsequently protecting healthy cells of the body [12]. A protective role of membrane-bound CD59 in context with OA has been proven in a mouse model [16]. As mentioned earlier, angiogenesis has been linked to OA [17]. There are studies that support pro- [18] as well as anti- [19] angiogenetic properties of the CS. However, detailed cartilage-specific neovascularization analysis of the CS is needed.

### 1.3. Prolactin

The protein hormone PRL is primarily produced by lactotrophic cells of the anterior lobe of the pituitary gland and secreted in a circadian rhythm. Various sites of extrapituitary PRL production are also known, such as the reproductive organs (human decidua), the immune system (B and T lymphocytes), and the brain (hypothalamus) [20]. PRL is important in the development of the mammary gland during pregnancy and the maintenance of milk secretion post-partum [21]. Nevertheless, PRL is also produced in males and non-lactating females, and normal circulating levels between 1 and 20 ng/mL suggest various other important physiological functions [21]. PRL is a component of human synovial fluid [22] and has been proven to be chondroprotective by inhibiting apoptosis [23] or increasing cell viability, chondrogenic differentiation, and proteoglycan accumulation [24]. PRL has a molecular weight of 23 kDa, contains 199 amino acids, and can be cleaved by various proteases such as CatD [25], matrix metalloproteinases (MMP) −8, −13 [26], and bone morphogenetic protein 1 [27]. Cleavage by these proteases results in the generation of vasoinhibin isoforms. Vasoinhibin reduces joint inflammation, bone loss, angiogenesis, and vasopermeability in murine antigen-induced arthritis [28]. Isoforms in the range from 5 to 18 kDa have been recognized [26]. PRL, in contrast, can promote angiogenesis by direct actions on endothelial cells or by the stimulation of growth factors such as FGF and VEGF [29].

### 1.4. Cathepsin D

CatD is a lysosomal aspartic endopeptidase with its optimal proteolytic activity at the acidic milieu of pH-value 3 [15]. Although its intra-lysosomal proteolytic property is broadly known [30], the cytosol and extracellular functions in a neutral pH are of great interest. CatD is known to cleave serum proteins such as complement protein C5 [15] as well as PRL [25], generating their split fragments C5a and vasoinhibin, respectively. Also, the cleavage of PRL for the generation of vasoinhibin under physiological conditions has been reported [31]. CatD can cleave aggrecan in a pH range of 5.2–6.5, thereby leading to a disintegration of the cartilage extracellular matrix [32]. At neutral pH, CatD has also shown that proteoglycan subunits can be degraded considerably. Even though the role of extracellular matrix degradation in neutral pH gets controversially discussed [33], the higher CatD activity in synovial fluid than in the serum of the OA patients [34] could still be responsible for the OA status of the patients. CatD-type enzyme activity was two to three-fold higher in ulcerated or yellowish articular cartilage from patients with primary OA compared with human cartilages of controls [35]. Hence, there is nonetheless importance of studying the role of CatD in joint tissue at neutral pH. Besides its proteolytic activity, the “ligand-like” additional function of Cat D in its zymogen status has been discussed [36,37]. Furthermore, both the pro- and anti-apoptotic effects of CatD have been discussed in a review article [38]. Therefore, CatD could directly or indirectly influence the chondrocytes in the pathogenesis of OA.

## 2. Methods and Materials

### 2.1. Isolation of the Chondrocytes and Cell-Culture

This study, with experiments using human-derived tissues, was approved by the Ethical Committee of the Bavarian Medical Association, No. 17029 (Approval date: 8 August 2017). Human cartilage tissue was derived from joint debridement material harvested during joint replacement operations in Nuremberg General Hospital. The donors were males and females between 19 and 75 years old with an average age of 42.2 years old. The written consent of the donors was taken prior to the tissue derivation. The donor tissue was rinsed in sterile phosphate-buffered saline (PBS) (Carl Roth, Karlsruhe, Germany) with 1% gentamycin (Biochrom AG/Merck, Berlin, Germany) and 1% penicillin/streptomycin (Biochrom AG/Merck, Berlin, Germany). After cutting the cartilage chips into small pieces (1–2 mm), the specimens were pre-digested for 1 h at 37 °C in a 2% pronase digestion medium (Dulbecco’s modified Eagle’s medium [DMEM]/Ham’s F12 1:1 w/o fetal calf serum (FCS) (Bio&Sell, Feucht, Germany) supplemented with 20 mg/mL pronase (Serva, Heidelberg, Germany), and incubated overnight at 37 °C with collagenase solution (0.1% collagenase NB5 Serva Electrophoresis GmbH, Heidelberg, Germany). The isolated cells were cultivated in growth medium consisting of DMEM/Ham’s F12 1:1 containing 10% fetal calf serum (FCS), 25 mg/mL ascorbic acid, 50 IU/mL streptomycin, 50 IU/mL penicillin, 2.5 μg/mL amphotericin B, essential amino acids (all: Biochrom AG/Merck, Berlin, Germany) and 0.05% trypsin/1.0 mM ethylenediaminetetraacetic acid (EDTA) (Biochrom AG / Merck, Berlin, Germany) were used to passage the chondrocytes. Chondrocytes until passage 5 were used for the experiments. The chondrosarcoma cell line Okayama University Medical School-27 (OUMS-27) was purchased (IFO 50488, JCRB Cell Bank, Ibaraki, Osaka, Japan) and cultivated in the above-mentioned growth medium. HUVEC (PromoCell GmbH, Heidelberg, Germany, Catalog Number: 12203) was also purchased and cultivated in endothelial cell growth medium (Cell Applications Inc., San Diego, CA, USA) containing 10% FCS, 50 IU/mL streptomycin, and 50 IU/mL penicillin.

### 2.2. Stimulation of Chondrocytes

A total of 15,000 cells/cm^2^ were cultivated for 48 h and subsequently serum-starved for 1 h in growth medium containing only 1% FCS before the stimulation. hAC, OUMS-27, or HUVEC were treated with various reagents, such as human serum-derived C5 (10 µg/mL; Merck KGaA, Darmstadt, Germany) and the recombinant proteins C5a (25 ng/mL; R&D Systems, Minneapolis, MN, USA), CatD (100 ng/mL; R&D Systems), and PRL (25 ng/mL; Abcam, Cambridge, UK) or the combination C5+CatD and PRL+CatD (same concentrations used as provided above) in growth medium containing only 1% FCS for 24 h and for 72 h.

### 2.3. Viability Staining of the Chondrocytes

The viability staining was performed with the stimulated chondrocytes, cultivated on Poly-L-lysin (Biochrom AG, Darmstadt, Germany) coated cover slides. The cells were incubated for 3 min at room temperature in a mixture of fluorescein diacetate (Sigma-Aldrich, Munich, Germany), which stains viable cells green, and propidiumiodide (Carl Roth GmbH, Karlsruhe, Germany), coloring dead cells red. The green and red fluorescence were visualized using confocal laser scanning microscopy (Leica TCS SPEII and DMi8, Wetzlar, Germany). The images of chondrocytes were analyzed using the Image Processing software ImageJ (US National Institutes of Health, Bethesda, MD, USA). The area covered by living cells was calculated in relation to the total area colonized by cells.

### 2.4. CyQUANT^®^ NF Cell Proliferation Assay

CyQUANT^®^ NF Cell Proliferation assay kit (Thermo Fisher Scientific, Eugene, OR, USA) was used to analyze the DNA content of chondrocytes by respective treatment at the endpoint of stimulation. The assay was performed according to the manufacturer’s protocol. A serial dilution of calf thymus DNA stock solution (1 mg/mL) (Sigma Aldrich, St. Louis, MO, USA) with tris(hydroxymethyl)aminomethane (TRIS)/EDTA buffer (10 mM TRIS (pH 8.0); 1 mM EDTA in H_2_O_deionized_) was used for the standard curve. After the stimulation, medium was removed, and the cells were washed carefully once with 1× Hank’s Balanced Salt Solution (HBSS) (Gibco, Loughborough, UK). HBSS was thoroughly removed, and 50 µL of the dye solution from the kit (1× HBSS + dye-binding solution 1:500) was applied to each cell-seeded well. Then, 25 µL of the standard dilutions were added with 25 µL of CyQuant dye solution (1× HBSS + dye-binding solution 1:250). After 60 min incubation at 37 °C, the fluorescence of each well was measured at 485Ex/530Em nm in a plate reader (Infinite M200 Pro, Tecan, Groedig, Austria).

### 2.5. The CellTiter-Blue^®^ Cell Viability Assay

The estimation of differences in metabolic activity of cells after stimulation was measured applying CellTiter-Blue^®^ Cell Viability Assay (Promega GmbH, Walldorf, Germany). A total of 4 h before the stimulation termination, 100 µL of the respective stimulation medium per well were mixed with 25 µL of Alamar blue solution for the incubation. Blue non-fluorescent resazurin contained in the incubation solution added to the growth medium penetrates the cells and is reduced by several intracellular (mitochondrial, cytosolic, and microsomal) redox enzymes into red and highly fluorescent resorufin depending on their cellular activity rate. The absorbance of each sample was measured in sextet at 570Ex/600Em nm in a plate reader.

### 2.6. Gene Expression Analysis

#### 2.6.1. RNA Isolation and cDNA Synthesis

The cell culture supernatant was removed after the stimulation, and the cells were rinsed with PBS before being lysed in 1:100 solution of β-Mercaptoethanol [Sigma-Aldrich, St. Louis, MO, USA] in RNeasy Lysis Puffer [Qiagen, Hilden, Germany]. RNA isolation mini kit [Qiagen, Hilden, Germany] was used to isolate total RNA using the manufacturer´s given instruction. Finally, RNA quantity and purity were assessed with the Nanodrop 1000 Spectralphotometer [Thermo Fischer Scientific, Erlangen, Germany]. cDNA synthesis was performed with Mastercycler [Eppendorf, Hamburg, Germany] using the isolated RNA.

#### 2.6.2. qPCR

Real-time detection polymerase chain reaction (qPCR) analyses were performed to obtain semi-quantitative gene expression data. The reference gene hypoxanthine guanine phosphoribosyl transferase and the specific primers from TaqMan^®^ Gene Expression Assays are listed in Table 1. A total of 1 µL aliquots of the derived cDNA was mixed to the TaqMan Gene Expression Master Mix solution (Applied Biosystems, Foster City, CA, USA) and water into a 10 µL reaction mixture to perform the TaqMan Gene Expression Assay. The assays were carried out in Applied Biosystems StepOnePlus^TM^ Real-Time PCR System (Life Technologies Holdings Private Limited, Singapore) following the manufacturer’s recommended protocol. Relative gene expression levels were normalized versus the reference gene and calculated with the 2-deltaCT method [39].

### 2.7. Immunofluorescence of Stimulated Primary Human Chondrocytes

The cell culture supernatant was removed after the stimulation experiment, and the stimulated chondrocytes on the coverslips were washed with TRIS buffered saline (TBS): 0.05 M TRIS, 0.015 M NaCl, pH 7.6 (Biochrom AG/Merck, Berlin, Germany). The cells were incubated with block solution: 5% Donkey serum (Chemicon, Temecula, CA, USA), 0.1% Triton X-100 (Sigma-Aldrich, St. Louis, MO, USA) in TBS for 20 min. The immunofluorescence staining procedure was performed using unconjugated primary and secondary antibodies (Table 2). Cell nuclei were counterstained using 4′,6- diamidino-2-phenylindole (DAPI), 0.1 µg/mL (Roche Diagnostics GmbH, Basel, Switzerland). F-actin filaments were stained using Phalloidin Alexa Fluor 488 or Phalloidin Alexa Fluor 633 (Life Technologies Corporation, Carlsbad, CA, USA). The images were taken using confocal laser scanning microscopy. The semi-quantitative analysis of protein expression in stained images was performed by measuring fluorescence intensity projected by cells using ImageJ (US National Institutes of Health, Bethesda, MD, USA) [40]. Corrected total cell fluorescence (CTCF) values were calculated for the analysis.

### 2.8. Statistical Analysis

GraphPad Prism, Version 8.1.4 (GraphPad Software, San Diego, CA, USA), was used for statistical analyses. Normalized data were expressed as the mean with standard deviation (mean ± SD). Differences between experimental groups were considered significant at *p* < 0.05 as determined by one sampled *t*-test (*). Shapiro-Wilk normality test was performed. ANOVA analysis was done using the Tukey’s post hoc Test (#). */# = *p* ≤ 0.05; **/## = *p* ≤ 0.01, ***/### = *p* ≤ 0.001. Grubb’s test was applied to identify and exclude the outliers.

## 3. Results

### 3.1. Viability Staining

All the applied stimulations (as described in Section 2.2) of the chondrocytes for 72 h did not show any significant effect on the viability of the cells (Figure S1).

### 3.2. CyQUANT^®^ NF Cell Proliferation Assay

**Figure 1:**
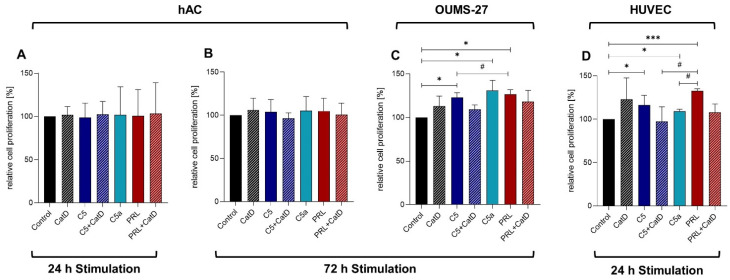
Graphic representation of the relative cell proliferation in response to 24 h (**A**) and 72 h (**B**) stimulation of human articular chondrocytes, 72 h stimulation of OUMS-27 (**C**), and 24 h stimulation of HUVEC (**D**) assessed by *CyQUANT^®^ NF Cell Proliferation* Assay. *n* = 3 (**A**), 5 (**B**), 3 (**C**), 5 (**D**) independent experiments with human articular chondrocytes from different donors. Mean with standard deviation. Control has been normalized to 100. One sample *t*-test with significance in relation to control (*). Mixed-effects analysis using post-hoc Tukey’s multiple comparisons (#). */# = *p* ≤ 0.05; *** = *p* ≤ 0.001.

No significant change in DNA content of primary human chondrocytes after any applied stimulation (C5a, CatD, C5+/−CatD, PRL+/−CatD, for details see Section 2.2) could be demonstrated after 24 h and 72 h using the CyQUANT^®^ NF Cell Proliferation assay (Figure 1A,B). However, significantly higher DNA amounts could be detected in response to C5, C5a, and PRL in OUMS-27 cells after 72 h of stimulation (Figure 1C) as well as in HUVEC after 24 h of stimulation (Figure 1D) in comparison to the respective unstimulated control groups. A significant increase in DNA was observed in the presence of PRL compared to the C5 stimulation in OUMS-27 cells and compared to the C5+CatD or C5a stimulation in HUVEC. Even though no significant changes were attained in primary human chondrocytes, 72 h stimulation showed a noticeable trend that represents similar effects seen in OUMS-27 (Figure 1B,C) as well as HUVEC (Figure 1B,D).

### 3.3. CellTiter-Blue^®^ Cell Viability Assay

CellTiter-Blue^®^ Cell Viability Assay showed no significant changes in metabolic activity and cell viability in primary human chondrocytes at any applied stimulation (Section 2.2) (Figure S2A). Only C5+CatD stimulation of the OUMS-27 showed a significant decrease in metabolic activity of the cells in comparison to the control group (Figure S2B).

### 3.4. Gene Expression

**Figure 2:**
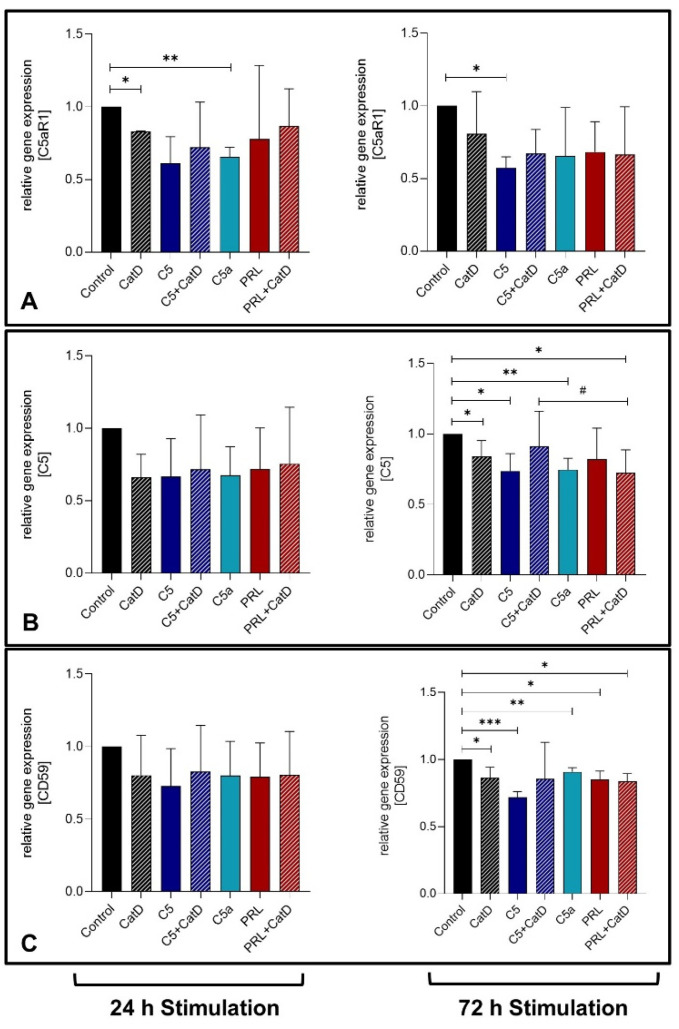
Graphic representation of relative chondrocytes gene expression of C5aR1 (**A**), C5 (**B**), and CD59 (**C**) after 24 h and 72 h of stimulation. *n* = 3 (24 h stimulation) and *n* = 5 (72 h stimulation) independent experiments with chondrocytes from different donors. Mean with standard deviation. Control has been normalized to 1. One sample *t*-test with significance in relation to control (*). Mixed-effects analysis using post-hoc Tukey’s multiple comparisons (#). */# = *p* ≤ 0.05; ** = *p* ≤ 0.01, *** = *p* ≤ 0.001.

In general, a trend was observed where all analyzed genes were downregulated under the influence of applied stimulations. CatD, as well as C5a treatment for 24 h, showed a significant suppression in C5aR1 gene expression in primary chondrocytes (Figure 2A). After 72 h, however, C5 stimulation also displayed a significant C5aR1 gene suppression. C5 gene expression was suppressed significantly after 72 h stimulation in all stimulated settings except for C5+CatD and PRL stimulation (Figure 2B). However, C5+CatD displayed rather higher C5 gene expression in chondrocytes compared to PRL+CatD. Similar to C5, the gene expression of CD59 after 72 h was also suppressed in all stimulated settings with significant values except in the C5+CatD stimulation in comparison to the control group (Figure 2C).

**Figure 3:**
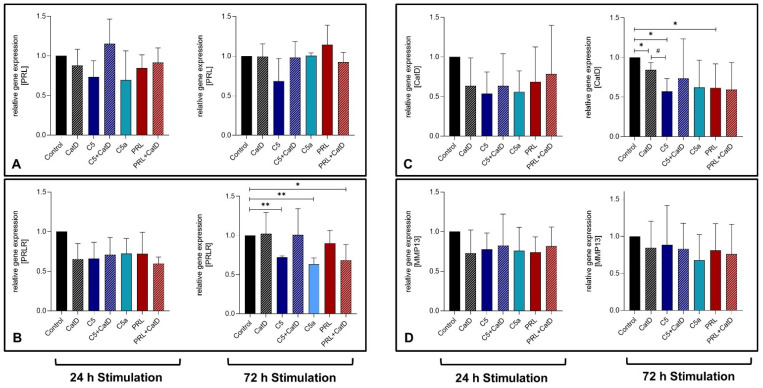
Graphic representation of relative chondrocytes gene expression of PRL (**A**), PRLR (**B**), CatD (**C**), and MMP-13 (**D**) after 24 h and 72 h of stimulation. *n* = 3 (24 h stimulation) and *n* = 5 (72 h stimulation) independent experiments with chondrocytes from different donors. Mean with standard deviation. Control has been normalized to 1. One sample *t*-test with significance in relation to control (*). Mixed-effects analysis using post-hoc Tukey’s multiple comparisons (#). */# = *p* ≤ 0.05, ** = *p* ≤ 0.01.

No significant effect could be detected in PRL gene expression under any stimulation (Figure 3A). C5, C5a and PRL in the presence of CatD could demonstrate suppression of the PRLR gene expression on a 72 h stimulation period (Figure 3B). Interestingly, during 72 h of stimulation, CatD is capable of significantly downregulating the CatD gene expression (Figure 3C). Besides, C5 and PRL both find common ground to suppress the CatD gene expression as well. No significant effect was detected in MMP-13 gene expression (Figure 3D).

### 3.5. Protein Synthesis

**Figure 4:**
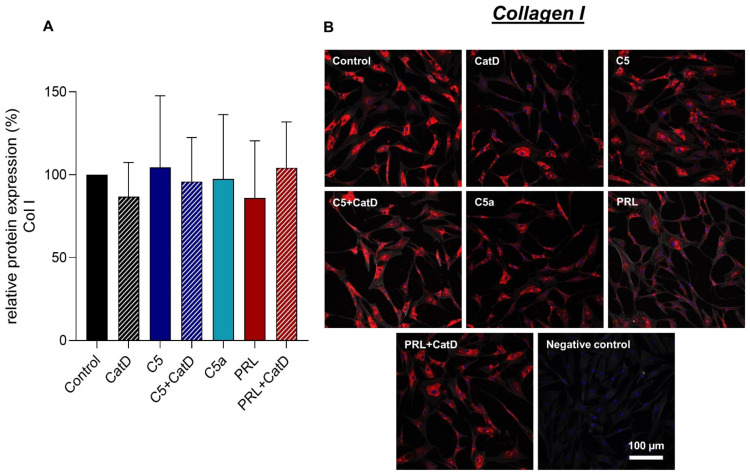
Chondrocytes protein expression of collagen type I after 72 h of stimulation and negative control of the staining. *n* = 4 independent experiments with chondrocytes from different donors. (**A**): Graphic representation of relative collagen type I protein fluorescence intensity, mean with standard deviation. Control has been normalized to 1. One sample *t*-test with significance in relation to control. Mixed-effects analysis using post-hoc Tukey’s multiple comparisons. (**B**): representative images of chondrocytes immunolabeled with collagen type I specific antibodies. Red (Cy3) = collagen type I, blue (DAPI) = cell nuclei, grey (Phalloidin Alexa 633) = actin cytoskeleton. Scale bar = 100 µm.

**Figure 5:**
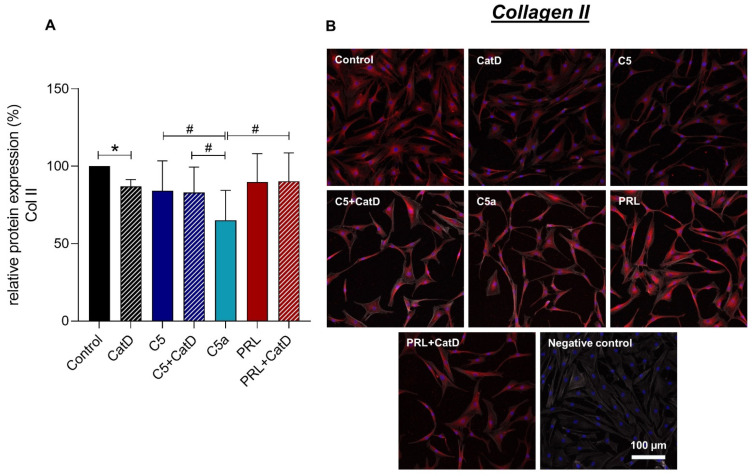
Chondrocytes protein expression of collagen type II after 72 h of stimulation and negative control of the staining. *n* = 3 independent experiments with chondrocytes from different donors. (**A**): Graphic representation of relative collagen type II protein fluorescence intensity, mean with standard deviation. Control has been normalized to 1. One sample *t*-test with significance in relation to control (*). Mixed-effects analysis using post-hoc Tukey’s multiple comparisons (#). */# = *p* ≤ 0.05. (**B**): representative images of chondrocytes immunolabeled with collagen type II specific antibodies. Red (Alexa Fluor 555) = collagen type II, blue (DAPI) = cell nuclei, grey (Phalloidin Alexa 633) = actin cytoskeleton. Scale bar = 100 µm.

**Figure 6:**
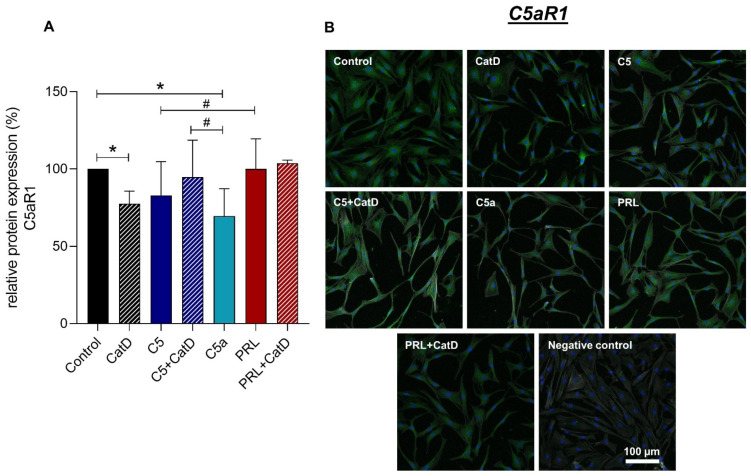
Chondrocytes protein expression of C5aR1 after 72 h of stimulation and negative control of the staining. *n* = 4 independent experiments with chondrocytes from different donors. (**A**): Graphic representation of relative C5aR1 protein fluorescence intensity, mean with standard deviation. Control has been normalized to 1. One sample *t*-test with significance in relation to control (*). Mixed-effects analysis using post-hoc Tukey’s multiple comparisons (#). */# = *p* ≤ 0.05. (**B**): representative images of chondrocytes immunolabeled with C5aR1 specific antibodies. Green (Alexa Fluor 488) = C5aR1, blue (DAPI) = cell nuclei, grey (Phalloidin Alexa 633) = Actin cytoskeleton. Scale bar = 100 µm.

**Figure 7:**
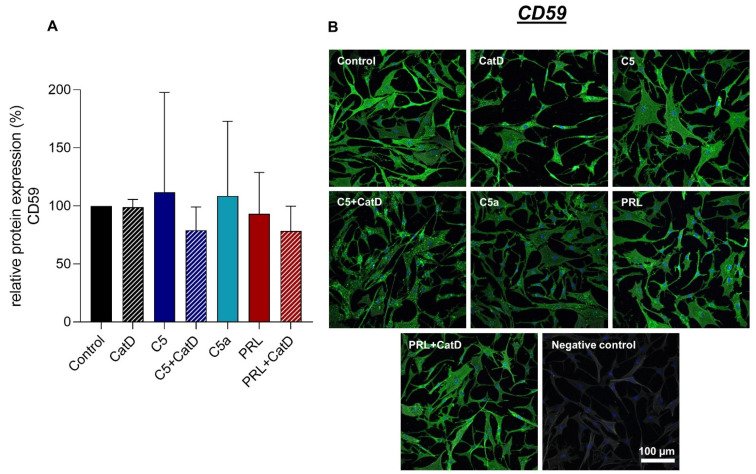
Chondrocytes protein expression of CD59 after 72 h of stimulation and negative control of the staining. *n* = 3 independent experiments with chondrocytes from different donors. (**A**): Graphic representation of relative CD59 protein fluorescence intensity, mean with standard deviation. Control has been normalized to 1. One sample *t*-test with significance in relation to control. Mixed-effects analysis using post-hoc Tukey’s multiple comparisons. (**B**): representative images of chondrocytes immunolabeled with CD59 specific antibodies. Green (Alexa Fluor 488) = CD59, blue (DAPI) = cell nuclei, grey (Phalloidin Alexa 633) = Actin cytoskeleton. Scale bar = 100 µm.

**Figure 8:**
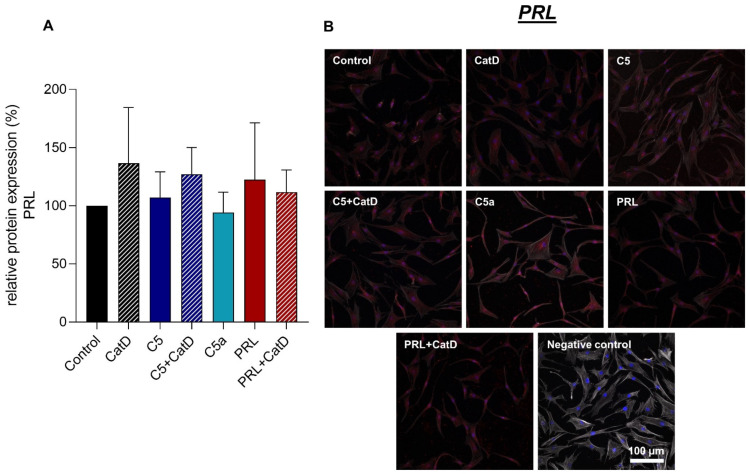
Chondrocytes protein expression of PRL after 72 h stimulation and negative control of the staining. *n* = 3 independent experiments with chondrocytes from different donors. (**A**): Graphic representation of relative PRL protein fluorescence intensity, mean with standard deviation. Control has been normalized to 1. One sample *t*-test with significance in relation to control. Mixed-effects analysis using post-hoc Tukey’s multiple comparisons. (**B**): representative images of chondrocytes immunolabeled with PRL specific antibodies. Red (Alexa Fluor 555) = PRL, blue (DAPI) = cell nuclei, grey (Phalloidin Alexa 633) = Actin cytoskeleton. Scale bar = 100 µm.

Intra- and extracellular protein expression of Col I, Col II, C5aR1, CD59, and PRL were evaluated using immunofluorescence staining (Figure 4, Figure 5, Figure 6, Figure 7 and Figure 8). An intracellular perinuclear Col I expression could be detected. Also, non-uniform distribution of Col I protein was found between the adjacent cells (Figure 6). Col II, C5aR1, CD59, and PRL expression were detected with homogenous cytoplasmic distribution in all stimulated and unstimulated chondrocytes (Figure 4, Figure 5, Figure 6, Figure 7 and Figure 8). Col II signal was impaired in CatD stimulated group in comparison to the control. C5a in comparison to C5+/−CatD or PRL+CatD also showed decreased protein synthesis of Col II (Figure 5). Furthermore, CatD lowered the C5aR1 protein synthesis compared to the control group. C5a stimulation of the hAC reduced C5aR1 protein synthesis in hAC compared to the control group as well as C5+CatD. PRL stimulation displayed higher C5aR1 protein synthesis compared to C5 stimulation (Figure 6). No significant results were obtained in respect to CD59 and PRL protein synthesis under applied stimulations (Figure 7 and Figure 8).

## 4. Discussion

Since the pituitary hormone PRL can be detected in the synovial fluid, expression of its receptor PRLR can be expected in the joint tissue [22,41]. It is so far known that PRL evokes a significant proliferative activity in human mesenchymal stem cells [22] and various other tissues [42]. Due to their cell proliferative property, PRL has been implicated in the initiation and progression of cancers [43]; antagonizing PRLR in certain tumors has therefore shown anti-proliferative effects on their cells [44].

The cell line OUMS-27, derived from chondrosarcoma tumor, could show a significantly higher proliferative effect of PRL in comparison to the non-stimulated group in the 72 h study. In contrast, no significant effect was observed in the primary chondrocytes after 72 h in our study. Primary chondrocytes, in comparison to tumor cells such as OUMS-27, have lower proliferation rate; therefore, the effect of the stimulations in hAC could probably be delayed. Since angiogenesis inside the cartilage is a characteristic of chronic stages in OA, and angiogenesis is generally associated with cell proliferation [45], we also analyzed the effect of the stimulation regime on the DNA content of HUVEC to estimate their proliferative response. PRL also demonstrated an increase in DNA, suggesting its proliferative effect in HUVEC, as reported earlier [46]. PRL displayed a more potent influence on DNA amounts suggesting cell proliferation in comparison to C5 or in comparison to C5+CatD in OUMS-27 and HUVEC, respectively. The proliferative effect of C5a but not that of C5 in endothelial cells has been studied before [47]. We could report enhanced DNA synthesis implicating cell proliferation in HUVEC under treatment with 10 µg/mL C5, compared to the above-cited study in which a human microvascular endothelial cell line (HMEC-1) was stimulated with 10 nM C5 (ca. 2 µg/mL) [47]. This shows either the phenotypical difference between the two endothelial cell types used in these different experiments or a concentration-dependent effect of C5 on the vascular cells. Since the physiological serum concentration of C5 is about 80 µg/mL [48] and in cases of systemic inflammation even higher, the proliferative effect of C5 in higher concentrations has yet to be determined. Despite non-significant results, CatD alone also showed a trend of enhanced DNA synthesis in all chondrocytes and HUVEC. This proliferative effect of CatD has been described before in fibroblast, endothelial cells, or even cancer cells [49,50,51] and shows relevance to be mentioned in this study as well.

None of the applied stimulations demonstrated impairment of the cell viability demonstrated by CellTiter-Blue^®^ Cell Viability Assay. The lower reduction of resazurin into resorufin in stimulated chondrocytes in comparison to the control group could either suggest a lower content of the cells or decreased intracellular or extracellular enzyme activity. Since no significant effect was detected in the hAC in this assay, this could be related to the non-significant proliferative effect on the hAC shown in the CyQUANT^®^ NF Cell Proliferation assay.

Recombinant C5a stimulation showed a clear C5aR1 gene suppression in primary chondrocytes after 24 h stimulation period. This effect has already been reported in the primary tenocytes [52]. This significant effect was also demonstrated in the protein synthesis level via semi-quantitative image analysis in a later time period. The lowered gene as well as protein expression of C5aR1 could be a self-protecting mechanism of the cells against an over-activation of the inflammatory activity mediated by C5aR1 activation. Likewise, CatD also contributed significantly to lowering C5aR1 gene expression in 24 h stimulation which was followed by a significant decrease in the protein level at the 72 h stimulation time. CatD lowered, furthermore, C5, as well as CD59 gene expression. The latter plays a crucial role in neutralizing the MAC formation. Synovial fluid C5b-9 level in early stages, as well as late stages of OA patients, is higher compared to healthy patients [16]. A decrease in CD59 expression could possibly compromise the complement regulatory capacity of chondrocytes, hence aggravating the OA status. C5 and C5a stimulation alone, as well as PRL+/−CatD, displayed similar responses lowering their CD59 gene expression in chondrocytes. Comparing PRL with C5 stimulation in chondrocytes, PRL stimulation also demonstrated higher C5aR1 protein synthesis in comparison to C5 stimulation. This effect could rather contradict the hitherto known chondroprotective role of PRL. Also, PRL with CatD suppressed C5 gene expression in chondrocytes. Even though no significant effect at the gene or protein level could be displayed in terms of PRL synthesis under the applied stimulations, PRLR, however, was downregulated by C5 as well as C5a stimulation and PRL in combination with CatD. This could also be genuine crosstalk where an over activation of the CS could lead to a downregulation of PRL/PRLR response and vice versa. As a versatile hormone or a cytokine, PRL via PRLR actively demonstrates its role in immune and inflammatory response, as discussed in a review paper by Yu-Lee et al., 2002 [42]. This immune aspect of PRL/PRLR signaling of CS is novel. Also, CatD has been associated with OA and other inflammatory diseases; the downregulation of CatD gene expression under PRL influence could rather be discussed as a chondroprotective aspect of PRL. The downregulation of the CatD gene was also triggered by C5, as well as CatD itself. MMP-8 and 13 are expressed at higher levels by OA chondrocytes than healthy chondrocytes [53]. MMP-1, -3, -8, -13 were described to be detected in the degenerated superficial layer of arthritic tissue [8]. In our study, no significant changes in gene expression of MMP-13 were detected under the applied stimulations.

Col I immunostaining in the chondrocytes displayed perinuclear distribution of the protein in chondrocytes. Overall the stimulated cells display a trend of rather decreased Col I protein expression. In contrast, Col II shows a homogenous intracellular as well as intercellular protein distribution. Ogueta et al., 2002 showed that human mesenchymal stem cells, which undergo chondrogenic differentiation, PRL stimulation show increased Col II as well as proteoglycan synthesis [22]. An induction in Col II protein synthesis in hAC compared to the control group could not be demonstrated in our study. However, C5a treated chondrocytes demonstrated impaired Col II protein synthesis in comparison to control, C5 +CatD, or PRL+CatD. Also, CatD demonstrated an impairment of Col II protein synthesis compared to the control group. Hence, both stimulations might indicate chondrodegeneration. It has to be taken into consideration as a limitation of the study that all effects observed during stimulation are dependent on time and concentration and could vary under different conditions. In the future, also intracellular regulatory effects via signal cascades have to be studied to understand the crosslinking between the two studied humoral systems.

## 5. Conclusions

C5, its cleavage product C5a, and the hormone PRL induced cell growth in OUMS-27 as well as in HUVEC but not in hACs. The significant suppression of the C5 gene expression under the influence of PRL+CatD and that of CD59 via PRL+/−CatD and conversely a PRLR gene suppression via C5 or C5a stimulation shows a possible crosstalk between the CS and the PRL hormone system in hACs. In addition, CatD as well as C5, in contrast to PRL, directly mediate possible negative feedback of their own gene expression. Further characterization is necessary to evaluate whether OUMS-27 is a complementary model for the primary articular chondrocytes. 

## Figures and Tables

**Table 1:** Oligonucleotides used for qPCR analysis.

Recombinant Proteins	Assay-ID	Company	Amplicon Length
HPRT	Hs99999909_m1	Applied Biosystems, Foster City, CA, USA	100
C5aR1	Hs00704891_s1		68
C5	Hs01004342_m1		66
CD59	Hs00174141_m1		70
PRL	Hs00168730_m1		106
PRLR	Hs01061477_m1		107
CatD	Hs00157205_m1		103
MMP-13	Hs00233992_m1		91

**Table 2:** List of applied antibodies for the immunofluorescence staining.

**Primary Antibodies**	**Company**	**Type**	**Catalogue Number**
Col1	SouthernBiotech, Birmingham, AL, USA	Goat-anti-human	1310-01
Col2	Origene Technologies, Rockville, MD, USA	Rabbit-anti-human	R1039X
C5aR1	GeneTex, Eching, Germany	Mouse-anti-human	GTX74845
CD59	Bio-Rad, Feldkirchen, Germany	Mouse-anti-human	MCA1054GA
PRL	Abbexa, Cambridge, UK	Rabbit-anti-human	abx100284
**Secondary Antibodies**	**Company**	**Type**	
Cy3	Dianova GmbH, Hamburg, Germany	Donkey-anti-goat	705-165-147
Alexa Fluor 555	Life Technologies Corp., Carlsbad, CA, USA	Donkey-anti-rabbit	A-31572
Alexa Fluor 488	Invitrogen, Waltham, MA, USA	Donkey-anti-mouse	A21202

## Data Availability

The data presented in this study are available in this article and its supplementary materials.

## Supplementary Materials

The following are available online at https://www.mdpi.com/article/10.3390/cells11071134/s1, Figure S1: Representative images of live-dead staining after 72 h stimulation of the primary human articular chondrocytes with a graphical representation; Figure S2: Graphic representation of the relative metabolic activity in response to 72 h stimulation of human articular chondrocytes (A) and 72 h stimulation of OUMS-27 (B) assessed by CellTiter-Blue® Cell Viability Assay.

## Abbreviations

AP	Anaphylatoxin
C5aR1	C5a receptor
CatD	Cathepsin D
Col I/II	Collagen type I/II
CRP	Complement regulatory protein
CS	Complement system
EDTA	Ethylenediaminetetraacetic acid
FCS	Fetal calf serum
hAC	Human articular chondrocytes
HUVEC	Human umbilical vein endothelial cells
MAC	Membrane Attack Complex
MMP	Matrix metalloproteinase
OA	Osteoarthritis
OUMS-27	Okayama University Medical School-27
PRL	Prolactin
PRLR	Prolactin receptor
