# Supplementary material for: Complement Proteins C5/C5a, Cathepsin D and Prolactin in Chondrocytes: A Possible Crosstalk in the Pathogenesis of Osteoarthritis
Source: Cells. 2022 Mar 28;11(7):1134. doi: 10.3390/cells11071134 (PMC8997946; doi:10.3390/cells11071134)
Supplement: Supplementary file 1 [file cells-11-01134-s001.zip › cells-1611642-supplementary.pdf]

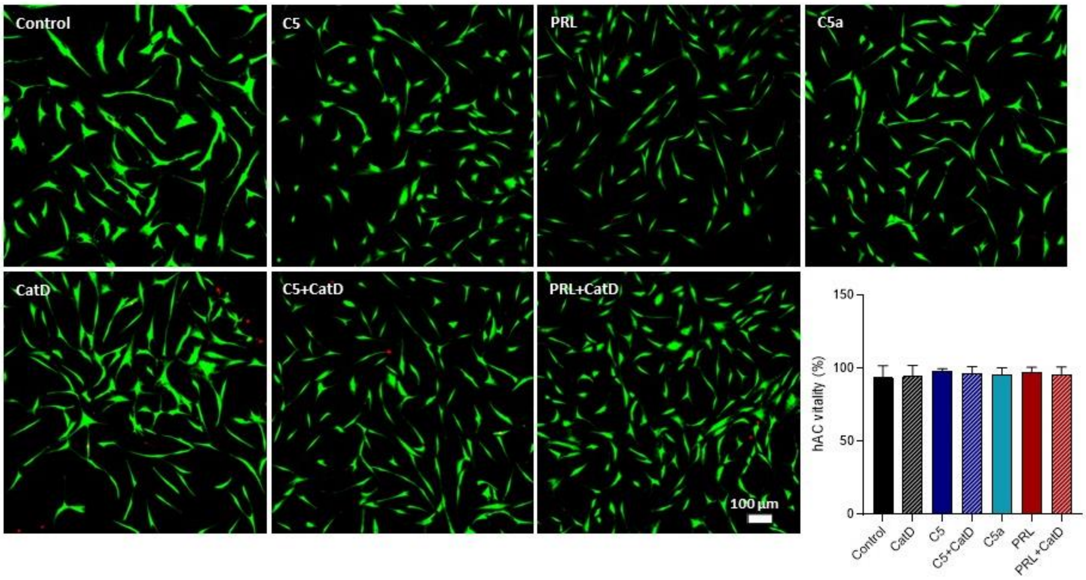

Figure S1: Representative images of live-dead staining after 72 h stimulation of the primary human articular chondrocytes with a graphical representation. Fluorescein diacetate (green: vital cells) and propidium iodide (red: dead cells). n = 3 independent experiments with chondrocytes from different donors. Mean with standard deviation. Values have been compared to the hypothetical value of 100% where all cells are vital after the experiment. One sample t-test with significance in relation to control. Mixed effects analysis using post Hoc Tukey´s multiple comparisons. Scale bar = 100 µm.

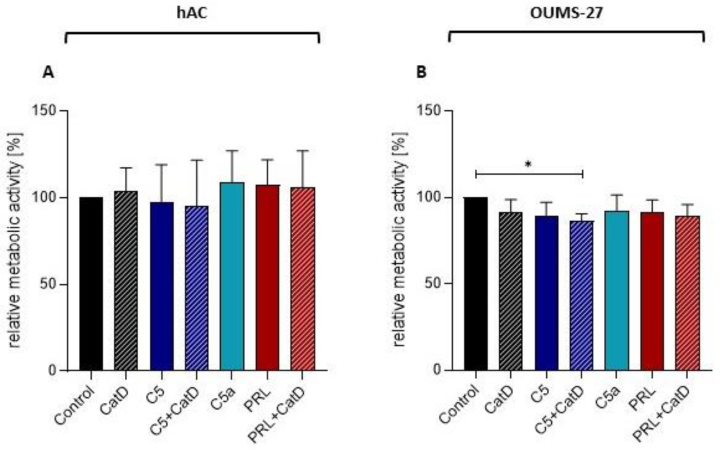

Figure S2: Graphic representation of the relative metabolic activity in response to 72 h stimulation of human articular chondrocytes (A) and 72 h stimulation of OUMS-27 (B) assessed by *CellTiter-Blue® Cell Viability Assay*. n = 5 (A), 3 (B) independent experiments with chondrocytes from different donors. Mean with standard deviation. Control has been normalized to 100. One sample t-test with significance in relation to control (*). * = $p \leq 0.05$.
